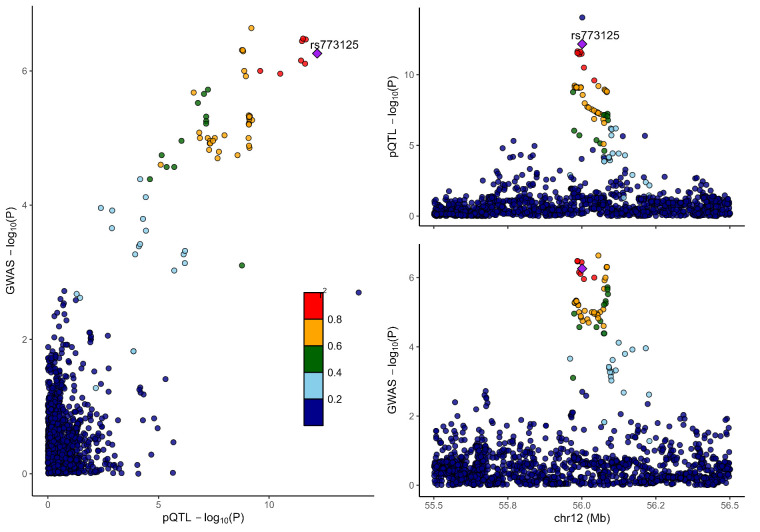# Potential therapeutic targets for multisite chronic pain: A proteome-wide Mendelian randomization study

**DOI:** 10.1097/MD.0000000000048579

**Published:** 2026-05-08

**Authors:** Jun Gao, JiaHao Liu

**Affiliations:** a Department of Neurosurgery, The First Hospital of Hunan University of Chinese Medicine, Changsha, Hunan Province, China; b Department of Urology, The First Affiliated Hospital of Nanchang University, Nanchang, Jiangxi Province, China.

**Keywords:** drug targets, Mendelian randomization, multisite chronic pain, plasma proteome

## Abstract

Multisite chronic pain (MCP) is a complex and increasingly prevalent health issue that significantly impairs quality of life. This study aims to identify potential therapeutic targets for MCP through a proteome-wide Mendelian randomization (MR) approach. We conducted a proteome-wide MR to explore the causal associations of plasma proteins with MCP. MCP used data from a genome-wide association study including 387,649 samples. We used protein data from UKB-PPP including 54,219 samples as the discovery analysis, and from Finngen as the replication analysis. Multiple follow-up analyses were used to investigate the potential function of the candidate proteins. Finally, druggability evaluation and phenome-wide MR analysis were used to assess the priority of these targets. We identified 11 plasma proteins significantly associated with MCP. Increased levels of LRP11, BCHE, DAG1, and SUOX exhibited protective effects, while LATS1, CEP170, SLC27A4, HEXIM1, ECM1, C8B, and MST1 increased MCP risk. After multiple validations, ECM1, C8B, LRP11, BCHE has the strongest convincing evidence. Besides, mediation analysis found 4 reliable combinations, revealed the role of plasma proteins in traits influencing MCP. Finally, druggability evaluation and phenome-wide MR indicated that C8B, and BCHE had the highest priority.

## 1. Introduction

Multisite Chronic Pain (MCP) is a prevalent health issue that has increased in impact in some regions in recent years.^[1,2]^ MCP is a phenotype of CP involving persistent or intermittent pain across multiple body sites. When severe, it can significantly impair an individual’s quality of life and have profound effects on daily functioning.^[3]^ The occurrence of MCP is associated with various factors, including genetic susceptibility, abnormalities in the immune and nervous systems, and lifestyle-related external factors such as obesity, physical activity, emotional disorders, and smoking.^[3–7]^ Compared to single-site pain, the complexity and widespread nature of MCP make it more challenging to manage. Patients often require comprehensive treatment approaches, including medication, physical therapy, and psychological support. However, existing treatments may be limited in effectiveness, providing only partial and temporary relief, or may have significant side effects and potential for addiction.^[4]^ This exacerbates the psychological and economic burdens on patients.

Proteomics, utilizing various sample types such as serum, plasma, and tissue, has become a crucial tool in CP research. Advances in technologies such as mass spectrometry have played a key role in identifying protein biomarkers associated with CP and elucidating the mechanisms underlying pain risk.^[8]^ Observational studies have identified some proteins related to MCP sensitivity, such as Panx1 and DUSP5.^[5,9,10]^ However, research on circulating proteins specifically related to MCP remains limited.

Conducting randomized controlled trials is the ideal method to study the impact of circulating proteins on CP, but such studies are challenging to implement. Since genetic variations are considered to be randomly allocated at birth, they are relatively independent of environmental influences. Mendelian Randomization (MR) analysis can serve as an alternative to randomized controlled trials, helping to determine causal relationships and reduce residual confounding and reverse causation common in traditional observational studies.^[11]^ We chose MR analysis to explore the causal relationship between circulating proteins and MCP, as it is considered the most effective method for addressing these questions.^[12]^ Protein quantitative trait loci (pQTLs) are genetic variations associated with plasma proteins identified through genome-wide association studies (GWAS). These variations are specific single nucleotide polymorphisms (SNPs) that regulate protein expression on chromosomes. PQTLs located within or near protein-coding genes are known as cis-pQTLs, while those located further away are called trans-pQTLs. PQTLs can be utilized in MR analysis to identify candidate causal proteins for MCP risk. MR analysis relies on 3 key assumptions: the relevance assumption, exclusion restriction assumption, and independence assumption.^[13]^ In the context of proteomics, these assumptions imply that the cis-pQTL should be strongly associated with the levels of its encoded protein, that the cis-pQTL influences the outcome exclusively through its effect on the plasma protein, and that the association between cis-pQTL and outcome were not affected by confounding factors. In this study, we conducted a proteome-wide MR analysis to systematically identify circulating proteins associated with MCP risk. Given that MCP is often linked to various factors, we also performed mediation MR to examine the connections between modifiable factors, plasma proteins, and MCP.

## 2. Materials and methods

### 2.1. Study design

**Figure 1.**
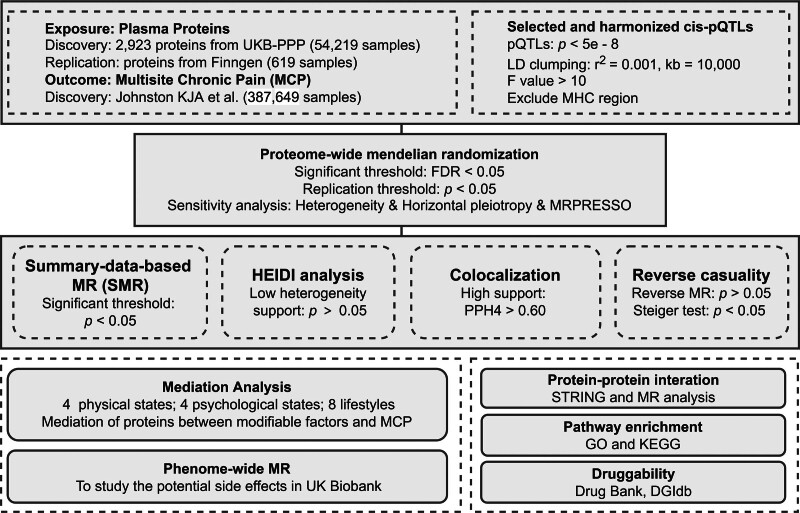
Overview of this study.

The study design is illustrated in Figure 1. We assessed the causal relationship between plasma proteins and MCP using proteome-wide MR analysis, complemented with protein replication analysis. To ensure the robustness of these associations, we utilized several validation techniques, including summary-data-based MR (SMR), the heterogeneity in dependent instruments (HEIDI) test, colocalization test, reverse MR, and Steiger filtering test. Additionally, we explored modifiable factors associated with MCP and examined whether plasma proteins mediate the causal impact of these factors on MCP through mediation MR analysis. For the identified proteins, downstream analyses were conducted included: Using mediation MR to explore protein pathways linking modifiable risk factors to MCP; protein–protein interaction (PPI); pathway enrichment; using phenome-wide MR to investigate the potential side effects of targets; druggability evaluation.

### 2.2. Study population and data source

All data used in this study were of European ethnic origin. The summary of statistical data on plasma protein genetic association was extracted from UKB-PPP (discovery analysis) and Finngen consortium (replication analysis). UKB-PPP contained 2923 proteins and included 54,219 participants.^[14]^ Large scale plasma protein study from Finngen consortium included 619 participants.^[15]^ Plasma proteins from 2 sources were both measured using the antibody-based proximity extension assay on the Olink platform and expressed in normalized protein expression units. Detailed descriptions of the above datasets can be found in original publications. Full summary statistics of MCP was obtained from a GWAS study containing 387,649 samples.^[16]^ MCP was defined as the total number of body sites where CP (at least 3 months duration) was reported, ranging from 0 to 7 sites. Individuals who indicated that they experienced CP “all over the body” were excluded. The screening criteria for SNPs can be obtained from the original research. Besides, the data source details of modifiable risk factor using in mediation MR can be found in Supplementary Table 1.

### 2.3. Proteome-wide MR analysis

We selected pQTLs based on the following criteria: pQTLs with genome-wide significant (*P* < 5× 10^−8^) association with any protein; independent pQTLs were determined by LD clumping with *r*^2^ <0.001 and a 10,000 kb window; the pQTLs were cis-acting and outside the major histocompatibility complex region (chr6, 26–34 Mb); and only pQTL with an *F*-statistic above 10 were included to ensure statistical validity (*F* = *R*^2^ × (N-2)/ (1-*R*^2^), R^2^ = 2 × EAF × (1-EAF) × beta^2^).^[17]^ In our study, we defined cis-pQTL as the leading SNP within 1 Mb of the transcription start site of the corresponding protein-coding gene. *F*-statistics were used to evaluate the statistical strength of each genetic instrument, and *R*^2^ indicated the proportion of variance in protein levels explained by each instrument.

We mainly employed the inverse-variance weighted (IVW) method to evaluate the causal relationship between plasma proteins and MCP. For plasma proteins with a single pQTL as the genetic instrument, we used the Wald ratio method. Multiple testing correction was applied using the false discovery rate (FDR) method. We then performed replication analysis (Finngen) by altering the plasma protein data, with a *P*-value <.05 in the replication MR considered statistically significant. When the number of cis-pQTL was sufficient, we conducted heterogeneity and horizontal pleiotropy tests, considering a *P*-value >.05 as a pass for these tests. Besides, MR pleiotropy residual sum and outlier (MRPRESSO) test was performed. We first focus on whether the *P*-value <.05 in the Main MR Results. Global Test *P*-value >.05 indicating no significant pleiotropy issues.

In this study, MR analysis was conducted using the “TwoSampleMR” package (version 0.5.6) and R software (version 4.3.2).^[18]^ The causal effect was evaluated by calculating the odds ratio with a 95% confidence interval (95% CI).

### 2.4. Reverse MR

To detect and remove potential reverse causality, we performed reverse MR for each significant protein. IV should satisfy the following criteria: demonstrating genome-wide significant association (*P* < 5 × 10^−8^). LD clumping with *r*^2^ <0.001 and a 10,000 kb window. The *F*-statistic of IV needs to be >10. We primarily used the IVW method to evaluate the causal relationship between MCP and plasma proteins. Furthermore, a key assumption in MR analysis is that the instrumental variable (IV) first affects circulating protein levels and subsequently influences the risk of MCP through these protein levels. To evaluate the directionality of each genetic instrument, the Steiger test was applied, with a *P*-value <.05 indicating that the IV has a stronger effect on the exposure.

### 2.5. Summary-data-based MR and HEIDI test

SMR combines MR analysis with QTL data, enabling the testing of associations between protein levels and traits of interest.^[19]^ The HEIDI test uses multiple SNPs to analyze and identify proteins associated with MCP risk, distinguishing those linked through a shared genetic variant rather than through genetic linkage^[19]^ We serve SMR as a method distinguished from 2-sample MR, can verify the reliability of proteome-wide MR results. All the SMR-based analyses mentioned above were conducted using SMR v1.3.1 (https://cnsgenomics.com/software/smr/). The analysis was carried out with default parameters: a *P*-value threshold of 5 × 10^−8^ was set to select the top associated QTLs for the SMR test, and a 2000 Kb window centered around the probe was used to select cis-QTLs.

### 2.6. Bayesian colocalization analysis

Bayesian colocalization analysis was employed to determine if the associations between the identified proteins and MCP resulted from linkage disequilibrium.^[20]^ Bayesian colocalization assigns posterior probabilities to 5 hypotheses, we focused on the posterior probability of hypothesis 3 and hypothesis 4 (PPH4). posterior probability of hypothesis 3 suggests that plasma proteins and MCP are significantly linked to SNP loci within a specific genomic region but are influenced by different causal variants. In contrast, PPH4 indicates that both traits are associated with the same causal variant in that region. A PPH4 value higher than 80% was regarded as strong evidence of a causal association and the presence of a shared causal variant. The colocalization analysis was conducted using the “coloc package” (version 5.2.3), and visualizations of colocalized regions were generated using the “LocusCompareR” package. Colocalization used the following parameters: p1 = 1 × 10^−4^, p2 = 1 × 10^−4^, p12 = 1 × 10^−5^.

### 2.7. Mediation MR analysis

We conducted mediation MR analysis to investigate whether circulating proteins mediate the causal relationship between modifiable risk factors and the incidence of MCP. First, we investigated 16 modifiable risk factors and divide into 3 categories, including: physical state: body fat percentage, body mass index, basal metabolic rate, and waist-to-hip ratio;^[16,21,22]^ psychological state: mood swings, anxiety disorders, depression, and subjective well-being;^[16,23]^ lifestyles:, leisure screen time, coffee consumption, cigarettes smoked per day, raw vegetable consumption, sleep duration, fresh fruit consumption, alcohol consumption, moderate-to-vigorous intensity physical activity during leisure time (MVPA).^[22,24–27]^ The sources of GWAS data for these risk factors are listed in the Supplementary Table 1. The criteria for selecting IVs are consistent with those previously described in reverse MR.

After 2-sample MR analysis, FDR *P*-value of <.05 in the IVW or Wald ratio method was considered significance for causal associations between modifiable risk factors, plasma protein, and MCP. Genetically determined causal effect of risk factors on plasma proteins was β1 and causal effect of plasma proteins on MCP was β2. Besides, causal effect of modifiable risk factors on MCP was c’. Mediation analysis was conducted using the “delta” method to calculate a 95% confidence interval (95% CI) and standard error. The Sobel test (https://quantpsy.org/sobel/sobel.htm) equation was applied to calculate the *P*-value of the mediation pathway strength. A *P*-value <.05 was considered indicative of a significant mediation effect.

### 2.8. Protein–protein interaction analysis

We utilized the STRING database to construct a PPI network for assessing PPIs (https://string-db.org/). Additionally, we performed a 2-sample MR analysis to investigate protein interactions. Cis-pQTLs were same as used in the proteome-wide MR, with the IVW and Wald ratio methods as the main approaches. FDR *P*-value <.05 was considered evidence of a causal relationship between the proteins.

### 2.9. Pathway enrichment

We performed pathway enrichment analysis on the selected proteins using the gene ontology database, which categorizes gene functions into Biological Process (BP), Cellular Component (CC), and Molecular Function (MF). Through this, we identified associations between plasma protein-coding genes and the CC, MF, and BP categories. Additionally, we used the Kyoto Encyclopedia of Genes and Genomes (KEGG) pathway database to analyze various biological pathways. The “clusterProfiler” package was employed to carry out the enrichment analysis.

### 2.10. Druggability evaluation and phenome-wide MR analysis

We assessed the therapeutic potential of the identified proteins by leveraging the DGIdb and DrugBank databases as resources.^[28,29]^ We aimed to retrieve the drug or component name, groups, actions and indication for each drug.

To investigate the potential side effects associated with these key targets, we analyzed summary statistics from traits within the UK Biobank cohort (N ≤408,961) as outcome measures. We employed cis-pQTLs of plasma proteins in initial analysis as IVs to perform a phenome-wide MR analysis, utilizing both the IVW and Wald ratio methods. FDR *P*-value of <.05 was deemed indicative of a statistically significant causal effect. Summary statistics were downloaded from the SAIGE GWAS (https://www.leelabsg.org/resources).^[30]^ To ensure adequate statistical power, we included only phenotypes with more than 500 cases in the phenome-wide MR analyses.

## 3. Results

### 3.1. Proteome-wide MR identified 11 circulating proteins causally affecting MCP susceptibility

**Figure 2.**
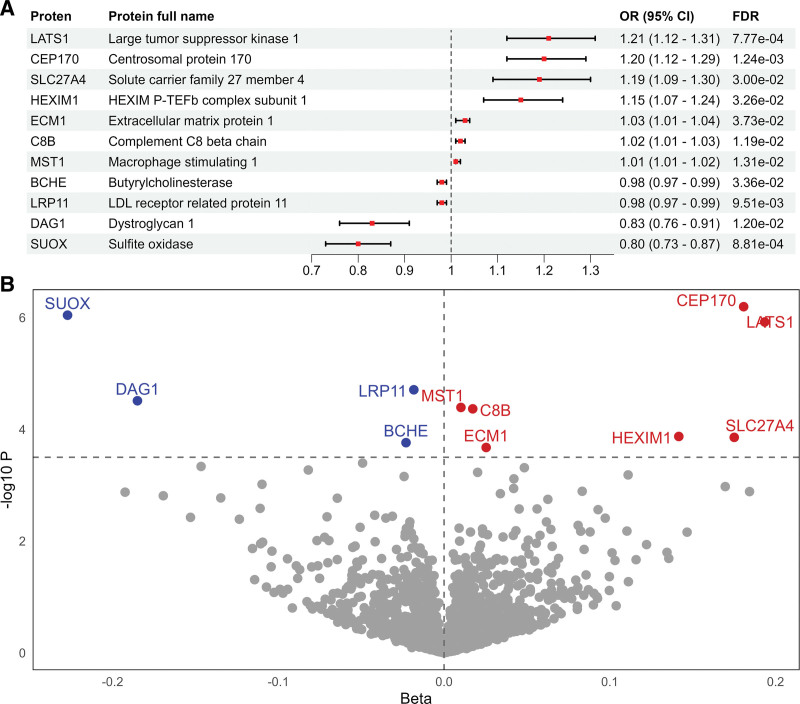
The MCP-associated plasma proteins. (A) The forest plot showed the protein’s full name, OR (95% CI), and FDR *P*-value. (B) The volcano plot showed the distribution of results from proteome-wide MR. CI = confidence interval, FDR = false discovery rate, MCP = multisite chronic pain, MR = Mendelian randomization, OR = odds ratio.

After screening 2923 proteins in the UKB-PPP, we identified 6145 cis-pQTLs associated with 1990 unique plasma proteins. All genetic instruments had *F*-statistics >10, demonstrating sufficient statistical power. IVs for significant proteins were presented in Supplementary Table 2. After FDR correction, a total of 11 proteins were identified (Fig. 2A and B). We found that genetically predicted higher levels of LATS1, CEP170, SLC27A4, HEXIM1, ECM1, C8B, and MST1 were associated with an increased risk of MCP, while higher levels of the serum LRP11, BCHE, DAG1, and SUOX were associated with a lower risk of MCP. There is no heterogeneity and pleiotropy found in these proteins. MRPRESSO also showed significance in Main MR Results and no pleiotropy in Global Test (Supplementary Table 3).

Reverse MR analysis extracted a total of 38 genetic instruments (Supplementary Table 6). No significant reverse causal relationship was found among these proteins (Supplementary Table 7). The Steiger filtering test further confirmed the directionality of all IVs for these causal association proteins.

In the replication stage, 5 proteins including ECM1, LRP11, C8B, MST1, and BCHE were successfully validated using plasma protein summary statistics from Finngen. Note that this is because we only successfully extracted cis-pQTL for these proteins. Our attempts to extract cis-pQTL for other proteins using a looser threshold failed. In addition, MR analyses using trans-pQTL for these proteins did not yield any significant results. The significance of these proteins that were not validated by any replication analysis needs to be treated with caution.

### 3.2. Colocalization analysis supported the causality of 5 proteins with MCP

To distinguish the causal relationship between genetically determined circulating protein levels and MCP from mere linkage disequilibrium, we conducted a colocalization analysis. Colocalization results were shown in Table 1. Among the 11 candidate proteins, ECM1, C8B, LRP11, LATS1, CEP170, and BCHE had PPH4 values exceeding 60%, providing strong evidence for an association with a shared causal variant. Visualization of colocalized regions is shown in Supplementary Figures 1,2,3,4,5,6,7,8,9,10 and 11, with details provided in Supplementary Table 4.

**Table 1:** Replication MR analysis, SMR analysis, reverse MR analyses, Steiger filtering test and Bayesian colocalization results of the identified plasma proteins.

Protein	*P*-value of replication	*P*-value of SMR	HEIDI test	Consistent directionality	Steiger test	Revers effect	PPH4	PPH3 + PPH4	Catagory
BCHE	2.85 × 10^−2^	1.53 × 10^−4^	0.89	Yes	TRUE	No	0.644	0.989	Tier 1
C8B	1.48 × 10^−5^	4.90 × 10^−5^	0.32	Yes	TRUE	No	0.063	0.998	Tier 1
CEP170	–	3.61 × 10^−6^	0.37	Yes	TRUE	No	0.704	1.000	Tier 3
DAG1	–	2.97 × 10^−4^	8.71 × 10^−4^	Yes	TRUE	No	0.585	0.920	Tier 3
ECM1	1.49 × 10^−10^	3.67 × 10^−11^	0.38	Yes	TRUE	No	0.715	0.992	Tier 1
HEXIM1	–	4.51 × 10^−4^	0.10	Yes	TRUE	No	0.001	1.000	Tier 3
LATS1	–	3.25 × 10^−5^	0.75	Yes	TRUE	No	0.002	1.000	Tier 3
LRP11	3.95 × 10^−6^	4.40 × 10^−6^	0.54	Yes	TRUE	No	0.744	0.986	Tier 1
MST1	2.43 × 10^−5^	2.69 × 10^−5^	1.66 × 10^−3^	Yes	TRUE	No	0.421	0.737	Tier 2
SLC27A4	–	9.52 × 10^−4^	0.64	Yes	TRUE	No	0.619	0.687	Tier 3
SUOX	–	4.73 × 10^−3^	NA	Yes	TRUE	No	0.946	1.000	Tier 3

HEIDI = heterogeneity in dependent instrument, MR = Mendelian randomization, PPH3 = posterior probability of hypothesis 3, PPH4 = posterior probability of hypothesis 4, SMR = summary-data-based mendelian randomization.

### 3.3. SMR analysis

To further validate our findings, we performed SMR and HEIDI tests on 11 proteins linked to MCP. All proteins showed *P*-values below .05, confirming they passed the SMR test. (Supplementary Table 5). The association directions of all causal proteins with MCP remain consistent with the proteome-wide MR results. Eight proteins also passed the HEIDI test. However, DAG1 and MST1 failed in HEIDI test; SUOX cannot perform a HEIDI test.

As shown in Table 1, drawing from the collective evidence, we classified these proteins into 3 distinct tiers.: Tier 1: 4 proteins (BCHE, C8B, ECM1, and LRP11) passed all tests. Tier 2: 1 proteins MST1 failed in the HEIDI test and/or colocalization analysis. Tier 3: 6 proteins (CEP170, DAG1, HEXIM1, LATS1, SLC27A4, and SUOX) lack additional replication analysis.

### 3.4. Mediation analysis found 4 reliable combinations

**Figure 3.**
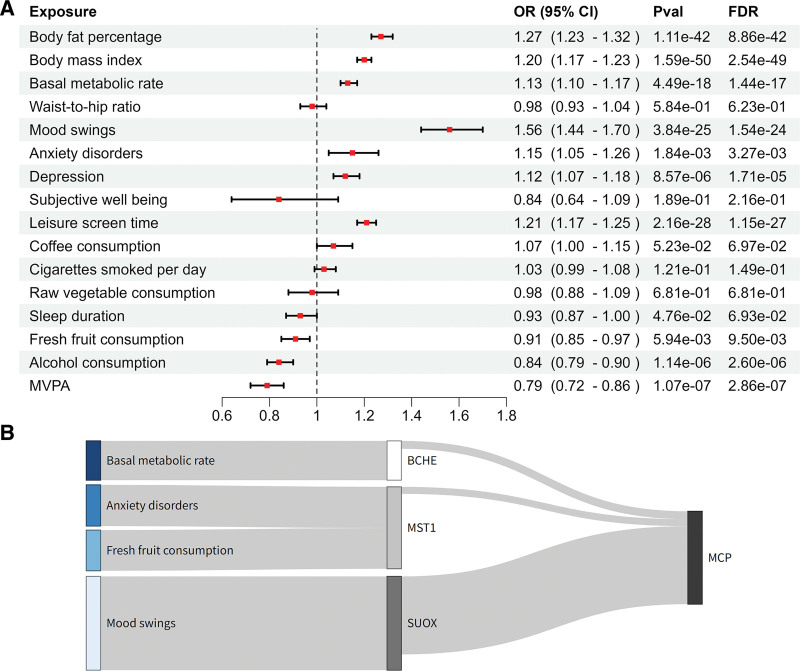
Mediation analysis results. (A) The forest map illustrates the causal relationship between risk factors and MCP; OR (95% CI), *P*-value, FDR *P*-value were also showed. (B) The sankey diagram displays 4 reliable pathways of risk factor-protein-MCP combinations. CI = confidence interval, FDR = false discovery rate, MCP = multisite chronic pain, OR = odds ratio.

Figure 3A revealed that 10 risk factors were associated with MCP. Genetically predicted higher levels of body fat percentage, body mass index, basal metabolic rate, mood swings, anxiety disorders, depression, leisure screen time, are associated with an increased risk of MCP, while higher genetically predicted fresh fruit consumption, alcohol consumption, MVPA levels are associated with a decreased risk of MCP. Most associations observed heterogeneity. Thus, a random effects model was used to adjust the IVW. However, horizontal pleiotropy was observed in the analysis of body fat percentage (*P* = .001), body mass index (*P* = .002) and alcohol consumption (*P* = .026). This suggests that IVs may affect MCP through factors beyond these risk factors. Horizontal pleiotropy may lead to bias in causal estimation, making the results of MR analysis less accurate. These results should be carefully evaluated. Genetic instruments and MR results of 16 phenotypes were shown in Supplementary Tables 8 and 9, respectively.

In the MR analyses of MCP-associated risk factors and plasma proteins, we used a significance threshold of FDR <0.05 to identify as many potential mediation signals as possible. Horizontal pleiotropy was only detected for the association between cigarettes smoked per day and LRP11 (*P* = .024). This approach resulted in the identification of 50 risk factor-protein association pairs. Mediation analysis finally found 10 pathways of modifiable factors-protein–MCP combinations with the *P*-value being <.05, and the direction of the total effect aligning with the mediated effect. However, because of pleiotropy was detected in c’ including: body fat percentage, body mass index and alcohol consumption, we consider these to be unreliable combinations and have 4 reliable combinations remaining. Sankey diagram was used to illustrate the mediating relationships among risk factors, plasma proteins, and MCP (Fig. 3B). The left nodes represent risk factors; the middle nodes represent plasma proteins, including BCHE, MST1, and SUOX; and the right node represents the outcome variable MCP. The width of the links corresponds to the strength of the mediation effect. Among the causal associations of these modifiable risk factors with MCP, 3 circulating proteins (BCHE, MST1 and SUOX) have significant mediating effects on the association between 4 risk factors (including anxiety disorders, basal metabolic rate, fresh fruit consumption and mood swings) and MCP risk. Among them, SUOX had the highest proportion of mediators in the association between mood swing and MCP (*P* = 8.03 × 10^−3^; proportion [95% CI]: 20.49% [15.90%−25.07%]). Supplementary Table 10 illustrated proportion of association between risk factor and MCP mediated by a circulating protein and details including proportion mediated and proportion 95% CI.

### 3.5. PPI and pathway enrichment

We conducted a PPI network analysis to explore the interactions among the 11 identified plasma proteins. We identified 3 pairs of interactions at 0.15 confidence score: LATS1 and MST1, MST1 and C8B, C8B and ECM1. However, after setting the threshold of the confidence score to 0.4, only LATS1 and MST1 can be detected. Next, we employed a 2-sample MR approach to investigate interactions among these 11 causal proteins and identified 5 significant associations with an FDR < 0.05, including: DAG1 and MST1, LATS1 and LRP11, LRP11 and LATS1, MST1 and DAG1, ECM1 and MST1(Supplementary Table 11 and Supplementary Fig. 13).

Additionally, we performed pathway enrichment analysis using gene ontology and KEGG to assess whether these identified plasma proteins were involved in specific biological pathways. (Supplementary Fig. 12). The top enriched pathways for BP included regulation of protein serine/threonine kinase activity, regulation of cyclin-dependent protein serine/threonine kinase activity, negative regulation of cyclin-dependent protein kinase activity, negative regulation of cyclin-dependent protein serine/threonine kinase activity. For the aspect of CC, these proteins were involved in collagen-containing extracellular matrix, organelle envelope lumen, contractile ring, nuclear envelope lumen. In terms of MF, these targets were enriched in extracellular matrix binding, laminin binding, very long-chain fatty acid-CoA ligase activity, fatty acid transmembrane transporter activity. However, no enriched pathways for KEGG were found.

### 3.6. ]Druggability evaluation and phenome-wide MR analysis of druggable proteins

We collected data from 2 drug databases to reveal the druggability. Five plasma proteins LATS1, SUOX, C8B, BCHE and DAG1 associated with MCP have been targeted for drug development (Supplementary Table 12). LATS1, C8B, and BCHE were targets for drugs approved. Notably, there is already approved drug targeting BCHE that have analgesic effects.

**Figure 4.**
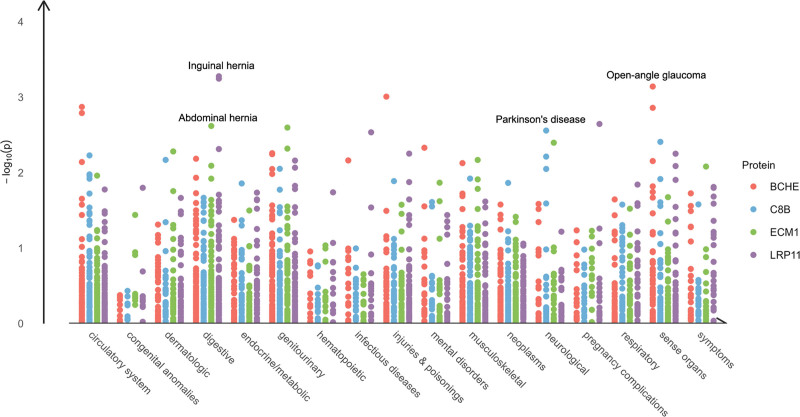
Phenome-wide MR results of 4 casual proteins with strongest convincing evidence. Vertical coordinate is -log_10_ (*P*-value), horizontal coordinate is disease category. MR = Mendelian randomization.

We evaluated whether the MCP-associated plasma proteins with the strongest evidence could influence other traits through a phenome-wide MR analysis. All results are presented in Supplementary Tables 13,14,15 and 16. Overall, after FDR correction, BCHE, C8B, ECM1, and LRP11 all showed no significant associations with any other phenotypes. Additionally, we labeled the most relevant phenotypes for each protein in Figure 4. Blood BCHE was negatively associated with open-angle glaucoma risk. Low C8B level correlated with high parkinson disease risk. ECM1 is mainly causally associated with inguinal hernia. Besides, LRP11 is negatively causally associated with abdominal hernia. Importantly, we emphasize that these associations did not meet the significance threshold after FDR correction, and therefore should be considered as invalid results that require further investigation in the future.

## 4. Discussion

In this proteome-wide MR study, we thoroughly explored the causal relationships between plasma proteins derived from UKB-PPP and MCP. In our primary proteome-wide analysis, we identified significant associations between plasma concentrations of 11 genetically determined proteins and MCP risk. Specifically, higher genetically determined levels of 7 proteins and lower levels of 4 proteins were linked to an increased risk of MCP. Sensitivity analyses, replication analysis, SMR analysis, colocalization test, and reverse causal association analyses on these plasma proteins increased the reliability of the proteome-wide MR results and divided these proteins into 3 categories. Our study identified 6 novel targets that have not been previously associated with the risk of MCP, including C8B, CEP170, DAG1, HEXIM1, SLC27A4 and SUOX. It should be noted that although the colocalization analysis of CEP170 showed statistical significance, it could not be replicated in this study due to the lack of GWAS data, and thus the association remains preliminary. Further verification for the role of CEP170 in MCP is necessary. Besides, we found 5 druggable proteins by druggability evaluation, in particular, a total of 40 drugs targeting BCHE. Phenome-wide MR did not find significant side effects, demonstrating the safety of these drug-forming targets.

We further explored these proteins in depth. Based on previous research, we categorized the 16 factors of interest into 3 groups: physical state, psychological state, and lifestyles. The risk factors of MCP were reassessed using MR and were used to assess the mediating role of plasma proteins, highlighting their roles in MCP pathogenesis. However, we found no significant causal association between the number of cigarettes smoked and coffee drinking with MCP. In addition, poorer psychological state was associated with more severe MCP, but there was no significant causal association between subjective well-being and less MCP. Notably, our KEGG analysis yielded no significantly enriched pathways. MCP is a highly systemic condition involving complex crosstalk between the central nervous system, immune responses, and psychological factors.^[31,32]^ Consequently, we think that the identified targets are likely distributed across a wide array of BPes rather than clustered within a single canonical signaling cascade.

Among these 11 notable proteins, BCHE, C8B, ECM1, and LRP11 had the strongest convincing evidence. Combined with the druggability assessment, BCHE and C8B were the most prioritized druggable targets. Butyrylcholinesterase (BCHE) belonging to the esterase group of enzymes, is a part of the serine hydrolase superfamily and serves a critical role in the hydrolysis of esters.^[33,34]^ BCHE has a broad substrate scope and lower acetylcholine catalytic efficiency.^[35]^ Individuals with BCHE deficiency typically remain asymptomatic, with the exception of an increased sensitivity to the muscle relaxants suxamethonium and mivacurium, both of which are BChE substrates used as myorelaxants.^[36]^ BCHE acts as an antidote in the body, especially in cases of nerve agent exposure, helping to minimize damage to the central and peripheral nervous systems from these toxic substances.^[37]^ Mouse experiments revealed that BCHE expression levels were significantly lower in samples with pulpitis-induced pain compared to normal samples.^[38]^ Notably, BCHE emerges as a promising target, as its therapeutic potential is clinically validated; several approved drugs that modulate this pathway, such as Rivastigmine and Donepezil (Supplementary Table 12), are already approved for Alzheimer disease, supporting the feasibility of repurposing such agents for pain management. Complement C8 beta chain (C8B) is an important component of the complement system, which is 1 of the 3 subunits of the C8 complex (C8A, C8B, and C8G) and plays a key role in the immune system, especially in the formation of the membrane attack complex.^[39]^ In previous studies, C8B has been shown to play an important role in psychosis, infectious and autoimmune diseases.^[40–42]^ However, no association between C8B and pain has been reported. Further epidemiologic and experimental studies are needed to determine our findings.

Extracellular matrix protein 1 (ECM1) plays an important role in the formation of the extracellular matrix, cell adhesion, signal transduction, as well as tissue differentiation and maturation.^[43]^ Many studies have shown that ECM1 is associated with autoimmune disease and cancer risk.^[44,45]^ In other MR analysis, ECM1 is causally associated with aging, asthma and topical dermatitis.^[46–48]^ LDL receptor related protein 11 (LRP11) is a member of the low-density lipoprotein receptor family consisting of transmembrane proteins.^[49]^ In various cancers including hepatocellular carcinoma, the expression of LRP11 was significantly higher in tumor tissues than in nontumor tissues, and the high expression of LRP11 was associated with poor prognosis of hepatocellular carcinoma.^[45,50]^ Besides, A recent proteome-wide MR study found that LRP11 was significantly associated with the risk of any migraine (*P* = 1.27 × 10^−6^, odds ratio (95% CI): 0.968 (0.955–0.981)).^[51]^ LRP11 also served as a first tier (strongest convincing evidence) protein and served as a protective factor for pain in that study. This means that LRP11 may be able to serve as a therapeutic target for many types of pain.

Our study has many strengths. The large sample sizes datasets used in this analysis enhance the statistical power and generalizability of our findings. Besides, we took many steps to avoid violating basic MR assumptions. We also performed a series of replication analyses, sensitivity analyses, reverse MR analyses, and colocalization test to ensure the stability of the results. Our results provide important insights into modifiable risk factors for MCP, which could inform future preventative strategies and clinical guidelines.

Our study has some shortcomings. Firstly, due to the limitations of the summary statistics data from Finngen, we were unable to extract cis-pQTL to perform replication analysis for several proteins, including CEP170, DAG1, HEXIM1, LATS1, SLC27A4, and SUOX. This may make the evidence for these plasma proteins underestimated. Secondly, we assessed the role of plasma proteins in MCP but could not estimate the levels of relevant proteins in other tissues especially in peripheral and central nervous system. Evaluating the role of protein levels in these tissues related to MCP could offer further insights into the disease’s pathogenesis. Thirdly, significant findings in Tier 3 that rely on a single SNP instrument (e.g., BCHE), despite having acceptable F-statistics, remain vulnerable to weak instrument bias and horizontal pleiotropy, which can lead to biased causal estimates. Additionally, our analysis utilized only cis-pQTL as IVs for plasma proteins; including trans-pQTL in future analyses may be more effective in identifying therapeutic targets for MCP. Lastly, this study was limited to European populations, and the applicability of these findings to other ancestries requires further validation.

## 5. Conclusion

In summary, our study identified 11 plasma proteins through proteome-wide MR that are causally linked to MCP. With further validation, these proteins could become novel targets for MCP therapy.

## Acknowledgments

The authors declare that there are no conflicts of interest or acknowledgments to report.

## Author contributions

**Conceptualization:** Jun Gao.

**Data curation:** Jun Gao.

**Formal analysis:** Jun Gao.

**Funding acquisition:** Jun Gao.

**Investigation:** Jun Gao.

**Methodology:** Jun Gao.

**Project administration:** Jun Gao.

**Resources:** Jun Gao.

**Software:** Jun Gao.

**Supervision:** Jun Gao.

**Validation:** Jun Gao.

**Visualization:** Jun Gao.

**Writing – original draft:** JiaHao Liu.

**Writing – review & editing:** JiaHao Liu.











**Figure:**
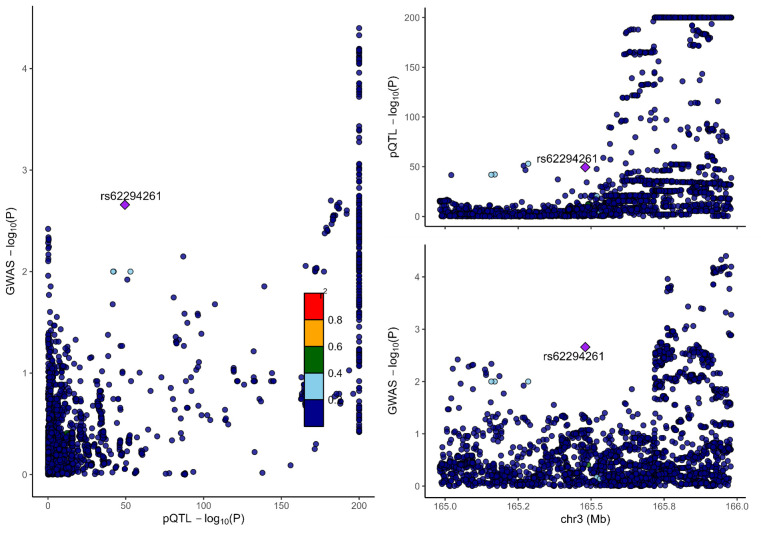














**Figure:**
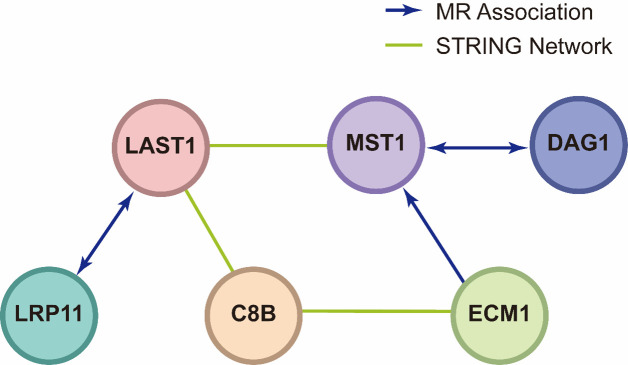


**Figure:**
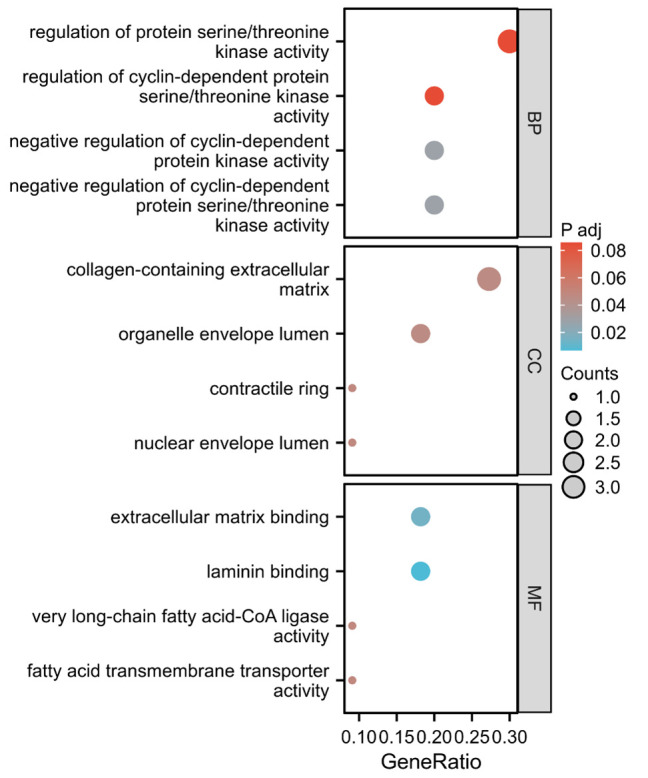












**Figure:**
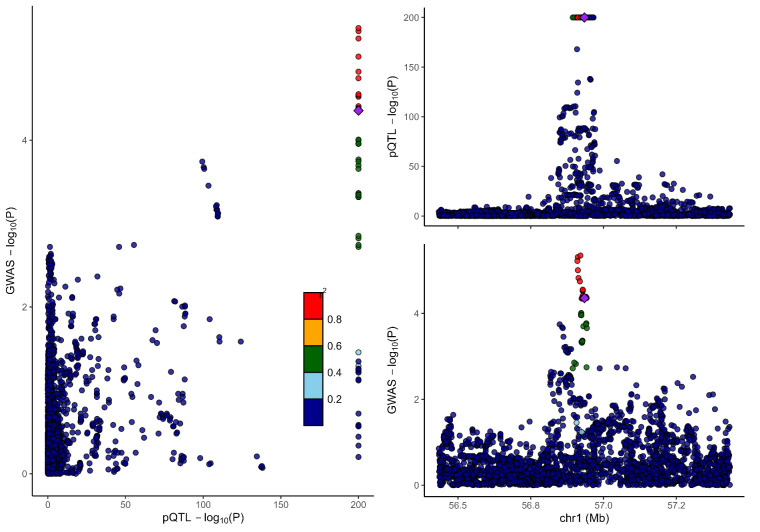


**Figure:**
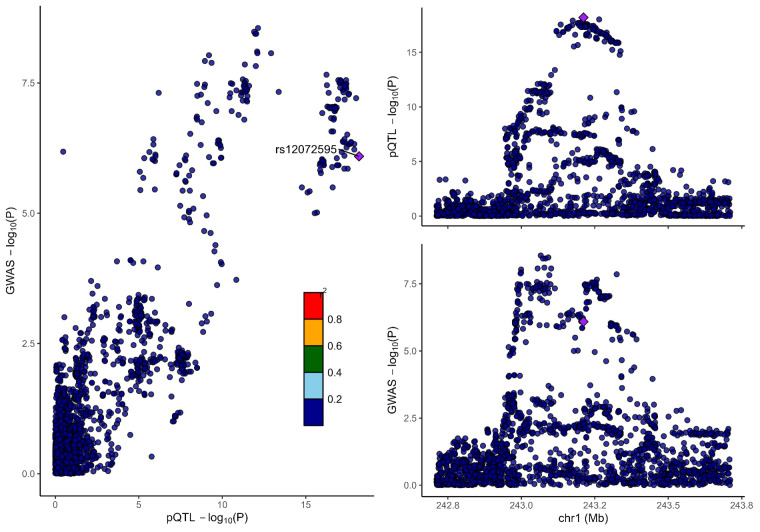


**Figure:**
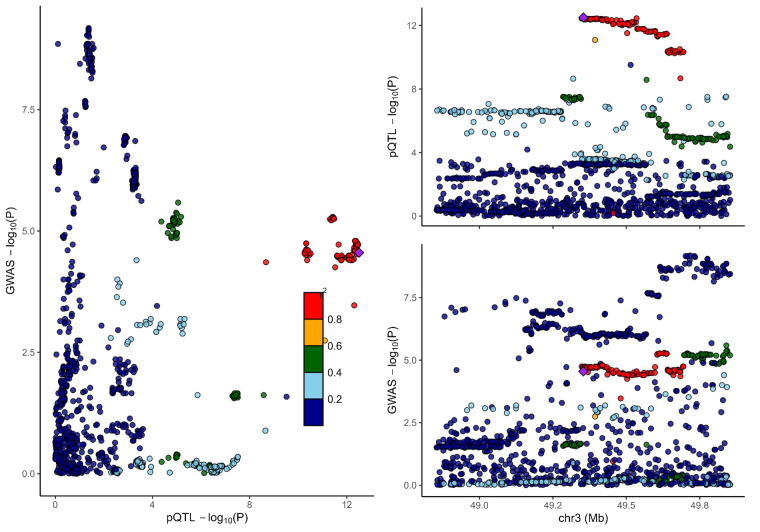


**Figure:**
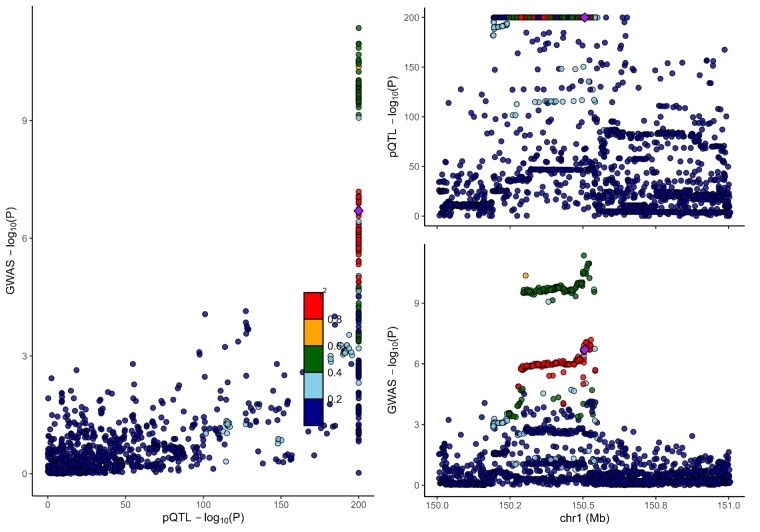


**Figure:**
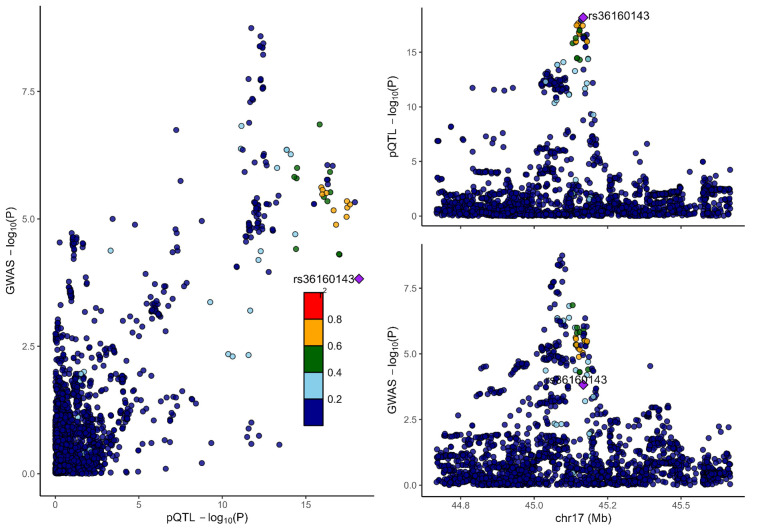


**Figure:**
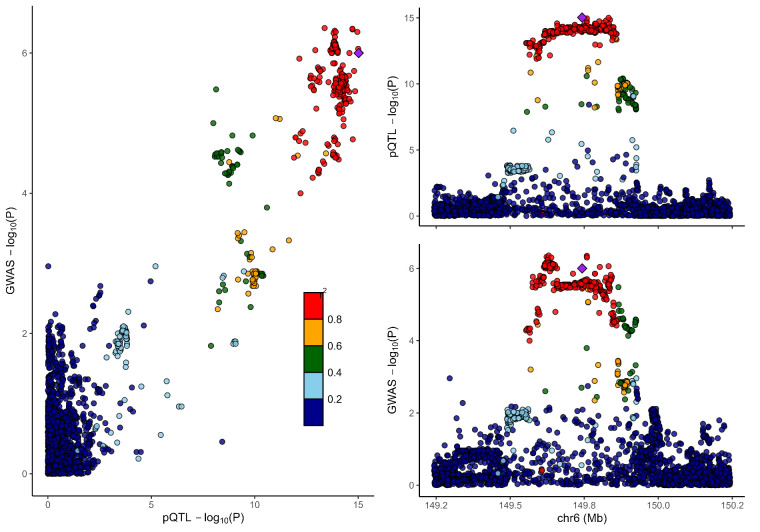


**Figure:**
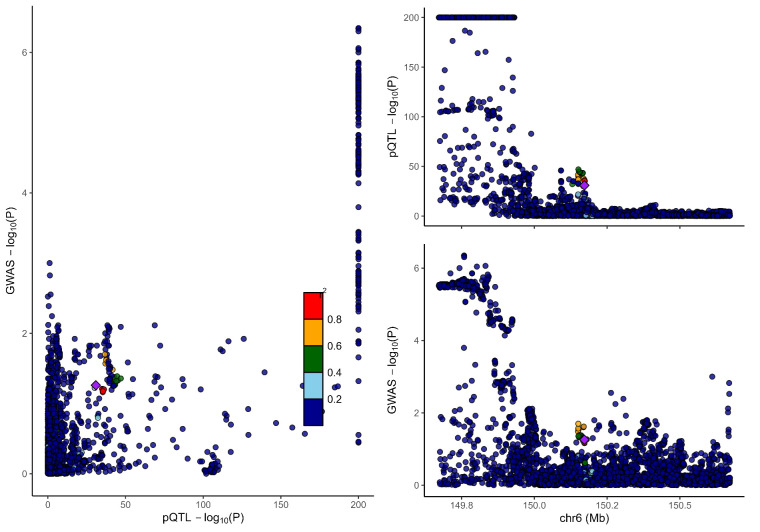


**Figure:**
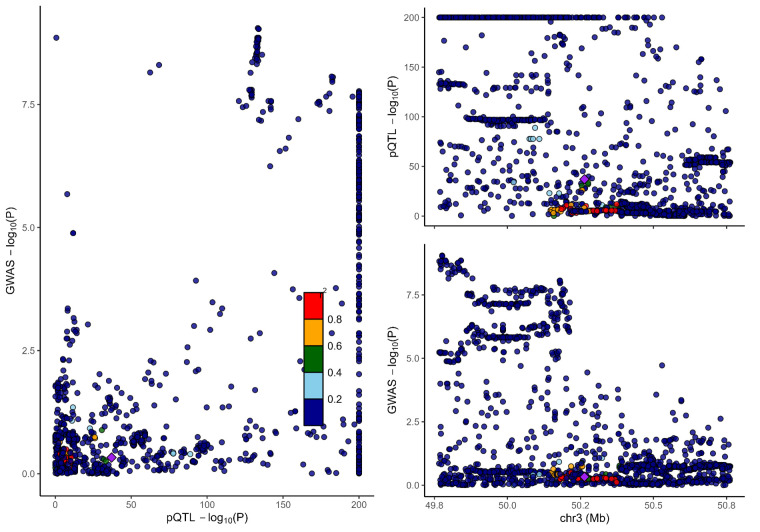


**Figure:**
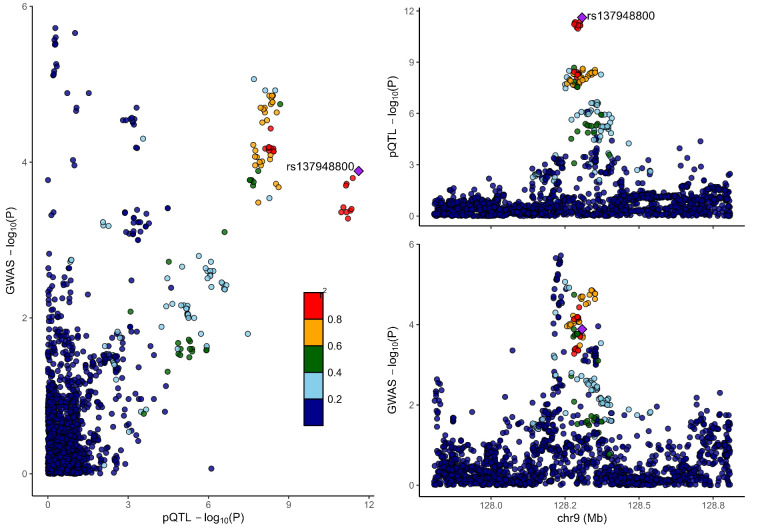


**Figure:**